# When meaning doesn’t matter, but location does: the effect of stimulus-hand proximity on conflict processing in the auditory modality

**DOI:** 10.1007/s00426-025-02171-8

**Published:** 2025-11-06

**Authors:** Aldo Sommer, Roman Liepelt, Rico Fischer

**Affiliations:** 1 Department of Psychology, University of Greifswald, Greifswald, Germany; 2 Department of General Psychology: Judgment, Decision Making, Action, Faculty of Psychology, University of Hagen (FernUniversität in Hagen), Hagen, Germany

**Keywords:** Stimulus-hand proximity, Auditory simon effect, Auditory stroop effect, Peri-personal space, Near-hand effect, Embodied cognition

## Abstract

Studies have shown that variations in stimulus-hand proximity alter conflict processing. Stimulus-response (S-R) conflict in visuo- and auditory Simon tasks increases when response hands are placed close (proximal) to the stimulus compared to when they are placed far (distal) from the stimulus. Conversely, a stimulus-stimulus (S-S) conflict in a classical visual Stroop paradigm was reduced in a proximal compared to a distal stimulus-hand condition. This suggests that stimulus-hand proximity may affect S-S and S-R conflict processing differently. However, it remains unclear whether a proximal stimulus-hand condition would also reduce the Stroop conflict in the auditory domain, where the task-irrelevant information requires pure semantic processing independent of the visual-spatial component of reading. The present study investigated the influence of stimulus-hand proximity on S-S and spatial S-R conflict processing in an auditory gender-categorization Stroop task (Experiments 1 and 2) and a Simon task (Experiment 3) by using the same stimulus materials in all experiments. The results consistently demonstrated that the auditory Stroop effect was unaffected by stimulus-hand proximity. This raises the question of the extent to which stimulus-hand proximity in previous demonstrations of reduced visual Stroop effects impacted semantic or rather visual-spatial processing. Finally, introducing a task-irrelevant spatial stimulus attribute and transforming the auditory Stroop task into an auditory Simon task increased interference in the proximal compared to the distal stimulus-hand condition. These findings suggest that response hands near visual and auditory stimuli seem to facilitate spatial feature processing.

## Introduction

In our daily lives, our cognitive system is often confronted with various kinds of conflicts, primarily caused by a mismatch between expected and actual outcomes. For example, when a basketball player fakes a shot, this creates a conflict for the defender, who expects a shot and must inhibit the related motor response. Typically, a coach would advise the defender to avoid focusing on the ball and instead concentrate on their opponent’s middle trunk movement (Meyer et al., 2022). In this example, reallocating spatial attention towards the relevant source of information helps the defender to avoid the conflict. Another piece of advice from the coach would probably be to maintain constant contact with the defender using one hand, allowing for rapid adaptation if their movement suddenly changes. From a cognitive perspective, the defender reallocates their spatial attention towards the relevant information by not only visually concentrating on another body part, but also by incorporating proprioceptive feedback from the opponent’s moving body through touch. This example highlights how our cognitive system continuously integrates multiple sensory input streams to guide our movement through space, and further demonstrates how cognitive processing can be modulated by sensory input from the environment and body position.

A prominent example of how multisensory integration impacts cognition is the near-hand effect (see Bridgeman & Tseng, 2011; Goodhew et al., 2015 for reviews), which describes that visual or auditory stimuli are processed differently when presented near to (proximal stimulus-hand condition) compared to far from the hands (distal stimulus-hand condition). In the domain of visual modalities, the placement of the hands near the stimuli has been shown to enhance target detection in a visual covert-orienting paradigm (Reed et al., 2006, 2018). Furthermore, Abrams et al. (2008) found that participants exhibited slower search rates in a visual search task, a reduction in inhibition of return (IOR), and a greater attentional blink in the proximal stimulus-hand condition compared to the distal condition. The authors interpreted these results as evidence of slowed attentional disengagement in the near-hand space. Thus, Abrams et al. (2008) suggested that objects appearing near the hands are perceived as potential candidates for manipulation and are therefore processed in greater detail. This in turn, slows attentional shifts within the near-hand space. The near-hand effect is also evident in the absence of hand (or foot) response (see Abrams et al., 2008), and even in the absence of hand visibility for the participant (Reed et al., 2006, 2018). It has further been demonstrated that merely imagining a near-hand posture slows down attentional shifts in a visual search paradigm (Davoli & Abrams, 2009), similar to the finding reported by Abrams et al. (2008). Furthermore, placing objects (Davoli & Brockmole, 2012) or the hand of a non-acting participant near the stimuli did not modulate task processing (Sun & Thomas, 2013, Experiments 1 & 2). Interestingly, however, task processing was modulated when the hand of a co-acting participant in a joint task was placed near the stimulus (Liepelt, 2014). Taken together, the evidence suggests that specifically the stimulus-hand proximity affects cognitive processing.

A promising mechanistic explanation for the outlined behavioral near-hand effect is the multimodal neuronal account first suggested by Reed and colleagues (2006). This account assumes that the proximity of objects to the hands facilitates the integration of multisensory information, which in turn leads to their attentional prioritization. The multimodal neuronal account is based on studies in single cell recordings of neurons in the posterior parietal and premotor cortex of macaque monkeys, (Fogassi et al., 1996; Graziano, 2001; Graziano et al., 1994, 1997, 1999). These neurons, referred to as bimodal or multimodal neurons, are assumed to form the basis for multisensory integration of vision, audition, and proprioception. It has been demonstrated that multimodal neurons exclusively respond to stimuli presented in close proximity to the monkey’s body (Fogassi et al., 1996; Graziano, 2001; Graziano et al., 1994, 1997, 1999; Rizzolatti et al., 1981), suggesting that they serve to evaluate spatial information based on a body-based or hand-centered coordinate system. Multisensory integration is thus employed by the cognitive system to interact with the environment by forming a representation of space by visual and/or auditory information relative to tactile and proprioceptive signals of the body (Brozzoli et al., 2012; Fang et al., 2024; Green & Angelaki, 2010; Stein & Stanford, 2008). If objects appear near the body (i.e., in peripersonal space), especially near the face and hands, they are mapped onto peripersonal networks, which in turn enable potential interactions through spatial body-object representations (Cléry et al., 2015; Hunley & Lourenco, 2018; van der Stoep et al., 2017). In the auditory domain, for example, bimodal neurons showed stronger activation when an auditory stimulus was presented in close proximity to the head (Graziano et al., 1999; Meredith & Allman, 2015). While the amplitude of the auditory stimulus might be used by the cognitive system to identify the location of the stimulus, tactile and visual information also seem to be used to encode its stimulus’ spatial location. It is important to note that this finding indicates the existence of bimodal (audio-tactile) and trimodal (audio-tactile-visual) neurons also in the context of multisensory integration in audio processing (Meredith & Allman, 2015). The finding of multimodal neurons that respond exclusively to stimuli presented in near-hand space may serve as evidence for a hand-centered attentional scope. This scope is thought to allocate attentional resources based on the hand’s position relative to objects, in preparation for potential interaction with such objects.

Importantly, stimulus-hand proximity has been shown to have opposing effects in conflict tasks. In conflict tasks (see also stimulus-response compatibility tasks; Kornblum, 1992, 1994; Kornblum et al., 1990), a conflict occurs when task-irrelevant information interferes with the selection or execution of the required response. This interference is behaviorally reflected in larger reaction times (RT) and error rates (ER) in conflict compared to non-conflict trials (i.e., when task-irrelevant information does not interfere with the required response). The interference is thought to arise, from an overlap in stimulus features, response features, or both (Kornblum, 1992, 1994; Kornblum et al., 1990). For example, in the Simon task, stimulus-response (S-R) conflict arises because the irrelevant spatial stimulus feature (i.e., the left or right location of the stimulus presentation) automatically activates the spatially corresponding left or right response. This leads to an S-R conflict when the cognitively controlled selection and execution of the required response to the stimulus’ identity (e.g., pressing left for a red stimulus) is spatially incompatible with the stimulus location (e.g., the red stimulus is presented on the right; Simon, 1969; Simon & Rudell, 1967; see Hommel, 2011 for a review). Manipulations of the stimulus-hand proximity in the Simon task typically led to larger interference effects when the response hands were located near the visually presented stimulus, rather than far from it (Liepelt & Fischer, 2016; Wang et al., 2014, 2018). Wang et al. (2014) suggested that placing the hand near a stimulus increases the perceived spatial similarity between the stimulus and the response location. They referred to the Gestalt principle, whereby elements in proximity are grouped together based on their spatial similarity (see Wagemans et al., 2012 for a review). Explaining the near-hand effect in the Simon task by such a facilitated coupling of spatial S-R features is also consistent with the multimodal neuronal account (Reed et al., 2006), in which the principle of multisensory integration relies on the spatial and temporal proximity of sensory inputs (van der Stoep et al., 2017). Therefore, the spatial proximity between the stimulus and the nearby hand increases the coupling of spatial S-R features. In other words, the spatial stimulus attribute (e.g., location) becomes more relevant, which may consequently facilitate a near-hand specific (pre-motor) response activation. While this benefits spatial compatible S-R relations (e.g., the identity of a stimulus presented on the right requires a right-side response), it leads to larger conflict in spatially incompatible S-R relations (e.g., the identity of a stimulus presented on the right requires a left-side response).

In contrast to spatial S-R conflict, opposing results regarding the stimulus-hand proximity effects have been observed in tasks involving a stimulus-stimulus (S-S) conflict (Davoli et al., 2010; Englert & Wentura, 2016; Weidler & Abrams, 2014). While a S-R conflict arises from an incompatible S-R relationship, a S-S conflict is thought to result from conflicting information at the stimulus level. For example, in a color-word Stroop task (MacLeod, 1991), the conflict arises when there is a mismatch between the task-relevant physical color of the word and its task-irrelevant semantic meaning (see also Kornblum, 1992, 1994; Kornblum et al., 1990). In visual S-S conflict tasks such as the Stroop task (Davoli et al., 2010) or the Flanker task (Englert & Wentura, 2016; Weidler & Abrams, 2014), placing the hand next to the target stimulus reduced the interference effects. Thus, the influence of stimulus-hand proximity on the processing of conflict stimuli appears to depend on the type of conflict: it enhances visual-spatial S-R conflict (Liepelt & Fischer, 2016; Wang et al., 2014, 2018; Yan et al., 2024), and reduces visual S-S conflict (Davoli et al., 2010; Weidler & Abrams, 2014).

Although there is ample evidence for the near-hand effect in visual conflict tasks (Davoli et al., 2010; Davoli & Brockmole, 2012; Englert & Wentura, 2016; Fischer & Liepelt, 2020; Liepelt & Fischer, 2016; Wang et al., 2014, 2018; Weidler & Abrams, 2014), little is known about it in auditory conflict tasks (Wang et al., 2021). To our knowledge, only one study has investigated the near-hand effect in an auditory Simon task so far. In this study, participants had to respond to the pitch of a tone by pressing either the left or the right button, while the tone was played randomly from a left or right speaker located in front of the participant (Wang et al., 2021). In line with results of studies on the near-hand effect in the visual domain (Liepelt & Fischer, 2016; Wang et al., 2014, 2018; Yan et al., 2024), Wang et al. (2021) also observed enhanced Simon effects in the near-hand space in their auditory setup. They explained these enhanced visual and auditory Simon effects in the near-hand space by a stronger spatial similarity between the stimulus and the response in the proximal stimulus-hand condition (Wang et al., 2014, 2021). Their finding of an enhanced auditory Simon effect in the near-hand space suggests that stimulus-hand proximity affects spatial S-R conflict processing in a similar way in auditory and visual modalities. However, it remains unclear whether this finding extends to other forms of conflict processing, such as S-S conflicts, which have been shown to be reduced in the near-hand space (Davoli et al., 2010; Davoli & Brockmole, 2012; Englert & Wentura, 2016; Weidler & Abrams, 2014).

### The present study

The present study aimed to extend the findings of previous studies on near-hand effects in a visual Stroop task with an S-S conflict (Davoli et al., 2010) to a setting with auditory stimuli. In Davoli et al.’s (2010) study, participants responded to the color of a word presented visually while ignoring its semantic content. An incongruent relationship between word color and meaning (e.g., the word ‘GREEN’ presented in red) typically leads to larger RT and ER than a congruent relationship (e.g., the word ‘GREEN’ presented in green), a phenomenon known as the Stroop effect (Stroop, 1935, 1938). The Stroop effect is primarily attributed to the process of automatic word reading interfering with the physical word color information (Appelbaum et al., 2009; see MacLeod, 1991, for a review). Davoli et al. (2010; see Experiments 2 and 3) observed that placing the hands near the stimuli reduced the Stroop effect. They suggested that stimulus-hand proximity attenuated semantic processing and thus reduced the interfering effects of automatic word reading. Several possibilities are conceivable:

Davoli et al. (2010) proposed an indirect effect of stimulus-hand proximity on semantic processing, suggesting a possible trade-off in favor of spatial processing and against semantic processing when reading words. Therefore, presenting a visual stimulus involves a spatial component, i.e., its spatial relationship with the nearby response hand. Visual stimuli close to the hands may trigger affordances due to the potential for object manipulation or action with the response hand. Therefore, the proximal stimulus-hand condition may facilitate the processing of spatial stimulus attributes (i.e., the spatial relationship between the stimulus and the response hand) at the expense of semantic processing. It is important to note that this assumption is based on the visual presentation of the stimulus (e.g., presenting words for reading).

Other explanations for reduced Stroop effects in the near-hand space are not restricted to visual stimulus processing. For example, cognitive control may be enhanced in the proximal compared to the distal stimulus-hand condition. Higher levels of cognitive control would increase the goal representation of performing the non-dominant color naming task and reduce the influence of the task-irrelevant reading process (Cohen et al., 1990). Arguments have been made for higher levels of cognitive control in near-hand space based on findings in task switching (Weidler & Abrams, 2014) and the numerical Simon task (Liepelt & Fischer, 2016). Finally, response hands placed near the visual stimulus may reveal a direct effect of some kind on semantic processing itself. This would not require any additional assumptions of a trade-off in favor of spatial processing at the cost of semantic processing as suggested by Davoli et al. (2010). However, Davoli et al.’s (2010) study was unable to differentiate between a direct effect on semantic processing and an indirect effect in the form of a bias towards spatial processing. It should be noted that that the modality of stimulus presentation is irrelevant to both the assumption of heightened cognitive control and impaired semantic processing.

In the present study, we modified the visual Stroop task into an auditory version and investigated whether stimulus-hand proximity influences the size of the Stroop interference. Participants had to identify the gender of the speaker’s voice (physical dimension) while ignoring the meaning of the spoken word (semantic dimension, i.e., woman or man). In an auditory task, semantic meaning is immediately available without the need for translation or subvocalization, enabling direct processing in working memory (Baddeley, 2003, 2012; Baddeley et al., 1988; Colle & Welsh, 1976). Therefore, in an auditory Stroop task no visual information needs to be translated into phonological codes, while the semantic dimension still drives the Stroop interference effect (Green & Barber, 1981, 1983). We derived the following predictions:

First, if the involvement of cognitive control is heightened and/or semantic processing is generally impaired in the near-hand space, the Stroop effect should be smaller in the proximal than in the distal stimulus-hand condition, regardless of the sensory modality of the stimulus presentation. This effect should also be evident in the auditory Stroop task.

Second, placing the hand near the stimuli may allow the cognitive system to encode stimulus attributes as stimulus-hand relations. However, a centrally presented auditory stimulus contains less spatial information (if any) than a visual stimulus in a visual-manual response mapping. Therefore, without a spatial attribute – i.e., a reference to the response hand – the trade-off between spatial and semantic processing, as assumed by Davoli et al. (2010), is not expected in the proximal stimulus-hand condition. Importantly, this assumption predicts no significant differences in auditory Stroop effects between proximal and distal stimulus-hand conditions.

Third, while this is not supported by previous findings in the visual domain (Davoli et al., 2010), it is possible that heightened attention in the near-hand space (Reed et al., 2006, 2007) can facilitate the processing of relevant and irrelevant task features. If both task-relevant and task-irrelevant features are more strongly activated, a higher threshold may be needed to determine the correct response. This would predict an even larger auditory Stroop effect in the proximal stimulus-hand condition compared to the distal stimulus-hand condition.

## Experiment 1

**Fig. 1:**
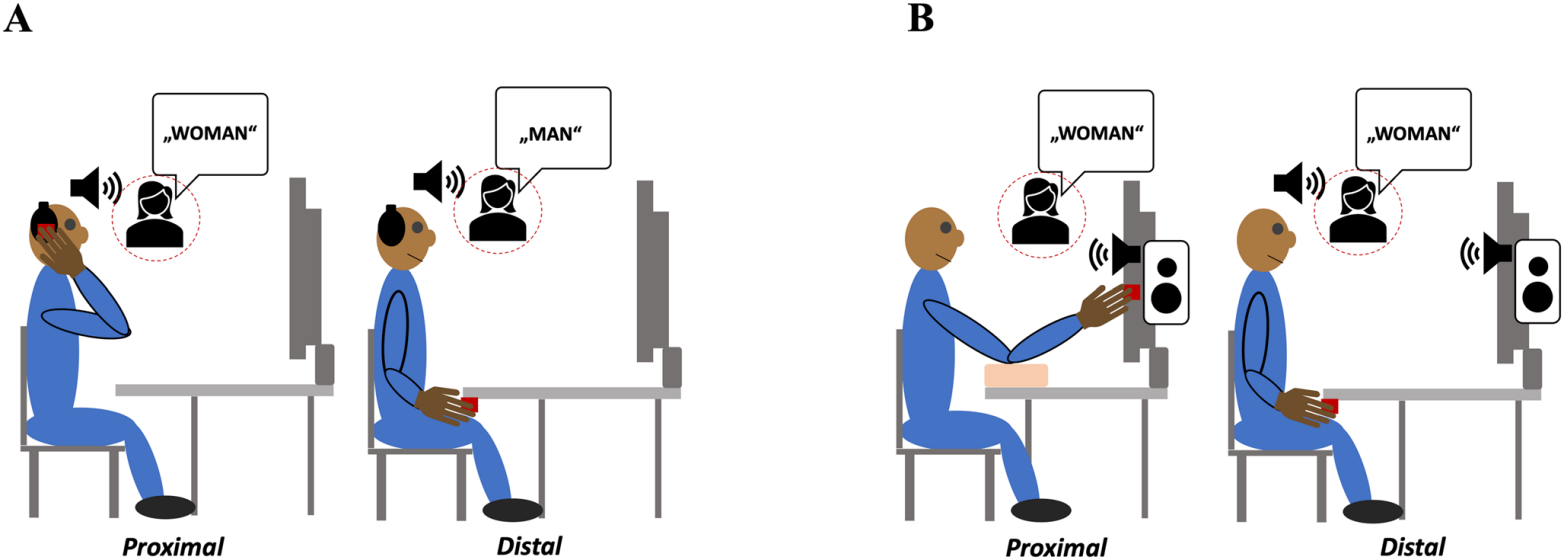
Schematic overview of the different hand positions. Panel A (Experiment 1): Participants responded with buttons attached to the headphones in the proximal stimulus-hand condition and with buttons located underneath the table in the distal stimulus-hand condition. Panel B (Experiments 2 and 3): Loudspeakers were attached to the left and right sides of the monitor. Participants responded with keys located on the loudspeaker in the proximal stimulus-hand condition. The location of the keys in the distal stimulus-hand condition was the same across all experiments.

To assess the possible explanations for the smaller visual Stroop effect reported by Davoli and colleagues (2010), participants were required to categorize the physical attribute of a speaker’s voice (i.e., male speaker or female speaker) presented via headphones. They were instructed to ignore the semantic meaning of the spoken German words *‘Mann’* (*man*) and *‘Frau’* (*woman*). The stimulus was considered congruent if the meaning of the spoken word (i.e., *man* or *woman*) matched the gender of the speaker’s voice (i.e., male or female voice) and incongruent if they did not (i.e., the word *man* spoken by a female voice, or vice versa). The Stroop effect was defined as the difference between incongruent and congruent trials. In the proximal stimulus-hand condition, stimulus-hand proximity was manipulated by placing participant’s hands on response buttons located on their headphones. In the distal stimulus-hand condition, participants operated the response buttons with their hands placed on their laps (see Fig. 1).

### Method

The design, hypotheses, and analysis plans of Experiment 1 were preregistered (AsPredicted #105257, https://aspredicted.org/G67_SBK).

#### Participants

An a priori power analysis (MorePower 6.0.4.) was carried out to estimate the required sample size for the experiment based on Davoli et al. (2010). According to the reported effect size of the Congruency × Hand position interaction in Experiment 3 (η^2^_p_ = 0.265) of the study by Davoli et al. (2010), a minimal sample size of 28 participants was recommended for the 2 × 2 effect of interest, with a power of 0.80, α = 0.05 (2-sided). To account for possible dropouts, 34 participants were recruited for the study. One participant had to be excluded because they finished the experiment prematurely (they stopped after the practice session). In addition, one participant had an error rate that was more than 3 SDs above the sample mean and was subsequently identified as an outlier and removed from the analysis. Thus, the final data set consisted of 32 participants (female: 23; left-handed: 3; age: 24 years ± 4 years, min: 18 years, max: 35 years). All participants reported normal hearing and stated that German was their first language. The study was conducted in accordance with the guidelines of the 1964 Declaration of Helsinki and those of the German Psychological Society. Participants provided informed consent prior to the experiment. Prior to the experimental session, each participant was informed of the study’s purpose and procedure. Participants were compensated with either course credits or a payment of €5.

#### Stimuli & apparatus

We used six computer-generated voices as stimuli, three modelled on female voices and three on male voices, originating from a study by Rothacher and colleagues (2020; see Experiment 1)^1^. Each male and female voice spoke the German word for *man* (‘*Mann’*) and *woman* (‘*Frau’*), resulting in 12 unique voice files. All stimuli were presented via QuietComfort over-ear headphones (Bose, Germany). The volume was standardized and noise cancellation deactivated. Participants responded using the index finger of each hand. In the proximal stimulus-hand condition, the left and right index fingers were located on response buttons attached to the headphones. In the distal stimulus-hand condition, the response keys were attached to the left and right sides of a wooden board positioned on the participant’s knees (see Fig. 1, panel A.). Stimulus presentation and data recording were controlled by the software PsychoPy^®^ (Version 2022.2.0) (Peirce et al., 2019) on a Windows 7 PC (Intel Core i5–6500 [3.2 GHz, 8 GB]).

#### Procedure

An auditory gender identification Stroop task (Green & Barber, 1981, 1983), previously applied by Rothacher and colleagues (2020), has been used. Participants were instructed to judge the speaker’s gender as fast and accurately as possible while ignoring the semantic meaning of the spoken word.

Each trial started with a fixation sign (i.e., *+*) at the center of the screen and remained there for a maximum of 3,000 ms. The target stimulus was presented after 1,000 ms of the fixation sign presentation and enabled the participants to respond. A response terminated the trial and feedback was shown. For correct trials, feedback was given by a blank screen for 300 ms (except for practice trials, here the German word for *correct*, *‘korrekt’*, was displayed after a correct response). For erroneous responses, the German words for *incorrect* (*‘falsch’*) were presented. The fixation sign was presented on a 17inch TFT monitor (physical resolution: 1280 × 1024 pixel; 60 Hz, Fujitsu Siemens, Germany). Viewing distance between the participant and the monitor was approximately 50 cm. All words and signs were presented in white color on a black background in the font Arial. A random interval with a blank screen between 100 ms and 1,000 ms (in the steps of 100 ms) was implemented before the next trial started.

The proximal and distal stimulus-hand conditions were conducted in separate experimental parts, containing one practice block and two test blocks for each hand position. The practice blocks consisted of 12 trials and each test block of 72 trials. The frequency of congruent and incongruent trials was distributed equally, with 50% in each block. Thus, practice trials excluded, participants performed 144 trials for each stimulus-hand condition.

The order in which the proximal and distal stimulus-hand conditions were presented was counterbalanced across participants. Thus, half of the participants started with the proximal stimulus-hand condition and then switched to the distal stimulus-hand condition, and vice versa. To further control for a possible S-R mapping effect, the response key assignments were also counterbalanced. Consequently, half of the participants responded to the female voice with their left index finger and to the male voice with their right index finger, while the other half of the participants responded with the reversed S-R mapping.

#### Data analysis

For the statistical analysis of the auditory Stroop task performance a 2 (Stimulus-hand proximity: proximal, distal) × 2 (Congruency: congruent, incongruent) within-subjects repeated measures analysis of variance (rmANOVA) was applied on RTs and ERs (mean percent errors). Analyses were conducted with the software RStudio (Version 2023.09.1, Posit Software, PBC). The rmANOVA was conducted by using the rstatix package (Version 0.7.2) (Kassambara, 2023). The results are presented in mean ± standard error of the mean. For RT analysis, error trials (4.7%) and RTs below 200 ms (0.0%) or above 3 SDs (1.2%) of the individual condition mean were removed prior to analysis.

### Results

**Fig. 2:**
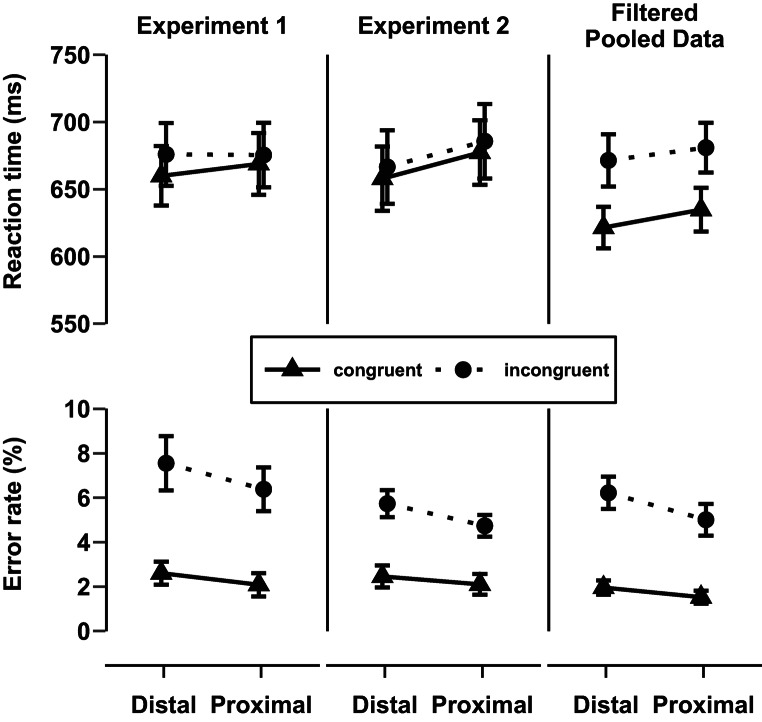
Mean reaction times and error rates from Experiments 1 and 2 as a function of stimulus-hand proximity and congruency. The right panel shows the pooled data from Experiments 1 and 2, filtered to include only voice files that produced a positive Stroop effect. Error bars represent standard errors of the mean.

Results are presented in Fig. 2.

#### RT and ER analyses

The rmANOVA with the within-subjects factors Congruency and Stimulus-hand proximity on RTs showed an overall Stroop Effect (11 ms ± 3 ms), indicated by a significant effect of the factor Congruency *F*(1, 31) = 4.24, *p* = .048, η^2^_p_ = 0.12. Thus, RTs were larger in incongruent trials (676 ms ± 17 ms) compared to congruent trials (664 ms ± 16 ms). However, neither did the hand position affect the overall RTs, *F*(1, 31) = 0.09, *p* = .766, η^2^_p_ < 0.01 (proximal: 672 ms ± 17 ms; distal: 668 ms ± 16 ms), nor was there an interaction between the factor Stimulus-hand proximity and Congruency *F*(1, 31) = 1.34, *p* = .256, η^2^_p_ = 0.04, indicating that the Stroop effect did not differ between the hand positions (proximal: 7 ms ± 5 ms, distal: 16 ms ± 5 ms).

The analogous ER analysis showed a similar pattern to the RT analysis. As expected, there was a significant overall Stroop Effect, *F*(1, 31) = 28.26, *p* < .001, η^2^_p_ = 0.48 (4.6% ± 0.5%). ERs were larger in incongruent trials (7.0% ± 0.8%) compared to congruent trials (2.3 ± 0.4%). Further, the ER differed according to hand position, *F*(1, 31) = 4.47, *p* = .043, η^2^_p_ = 0.13 (proximal: 4.2% ± 0.6%; distal: 5.1% ± 0.7%), thus participants committed less errors in the proximal compared to the distal stimulus-hand condition. However, most importantly for this study, the Stroop effect was not modulated by the hand position, as indicated by a lack of interaction between Stimulus-hand proximity and Congruency *F*(1, 31) = 0.54, *p* = .467, η^2^_p_ = 0.02 (Stroop effect = proximal: 4.3% ± 0.6%, distal: 5.1% ± 0.8%) (see Fig. 2, Experiment 1).

### Discussion

Experiment 1 was conducted to test the influence of stimulus-hand proximity on the auditory Stroop effect. We found evidence of a significant Stroop Effect in both RTs and ER. However, placing the response hands either proximal or distal to the auditory Stroop stimulus did not affect the size of the Stroop effect, as indicated by the absence of an interaction effect between Congruency and Stimulus-hand proximity. Therefore, our results did not provide evidence that the smaller Stroop effect observed by Davoli et al. (2010) for visual stimuli in the near-hand space could easily be transferred to auditory stimuli.

One possible reason for the absence of the near-hand effect in Experiment 1 may be that the participants’ hands were not visible in the proximal stimulus-hand condition. In this condition, the participant’s hands were positioned at the headphones they wore. This differs from previous studies, in which the participants’ hands were clearly visible within their visual field (Davoli et al., 2010; Ellinghaus et al., 2023; Fischer & Liepelt, 2020; Liepelt & Fischer, 2016; Wang et al., 2014, 2021). The study of Reed et al. (2006) has also shown that occluding the hands still produces a near-hand effect, albeit a smaller one than when the hand is visible. Furthermore, the cognitive representation of the space around the hand is primarily attributed to multisensory integration (Fogassi et al., 1992, 1996; Graziano, 2001; Graziano et al., 1994, 1997, 1999; Rizzolatti et al., 1981; see Cléry et al., 2015; di Pellegrino & Làdavas, 2015, for reviews), with a dominance of visual information overriding proprioceptive information in respective cortical brain areas (Makin et al., 2007). Therefore, the non-visibility of the hands in the proximal-stimulus hand condition in Experiment 1 might also be responsible for the absence of the near-hand effect.

## Experiment 2

In Experiment 2, the hands of the participants were placed visibly within their visual field in the proximal-stimulus hand condition. The auditory stimuli were presented via loudspeakers positioned to the left and right of the monitor (see Fig. 1, panel B). In this condition, participants used response buttons attached to the loudspeakers in front of them. In the distal stimulus-hand condition, the hand position of the participants remained the same as in Experiment 1. To improve Experiment 2 further, we recruited an equal number of male and female participants to avoid a potential gender bias in responses to the gender of the speaker’s voice.

### Method

The design, hypotheses, and analysis plans of Experiment 2 were preregistered (AsPredicted #122798, https://aspredicted.org/qtsg-vcrx.pdf).

#### Participants

A new sample of 40 participants was recruited. According to their self-reports, seven participants did not follow the experimenter’s instructions and either closed their eyes or gazed at a point on the wall. One further participant ended the experiment early, meaning that their data could not be used. These eight participants were therefore excluded from the present study, and another eight participants were recruited to achieve the desired sample size. Of the 40 participants in the resulting dataset, one committed an ER of more than 3 SDs of the sample mean. This participant was identified as an outlier and removed from further analyses. Thus, the final sample consisted of 39 participants (female: 19; left-handed: 4; age: 23 years ± 4 years, min: 18 years, max: 38 years). All participants reported sufficient knowledge of the German language and normal hearing. The study was performed in accordance with the guidelines of the 1964 Declaration of Helsinki and those of the German Psychological Society. Participants provided informed consent prior to the experiment. Before the experimental session began, each participant was informed of the study’s purpose and procedure. Participants were compensated with either course credits or a payment of €8.

#### Stimuli & apparatus

For Experiment 2, the same stimulus material and apparatus were used as in Experiment 1. The only difference was that the stimuli were presented via Z130 Compact 2.0 loudspeakers (Logitech, Switzerland) at a standardized volume. The loudspeakers were attached to the left and right sides of the monitor, with response keys attached on the outer side of each (see Fig. 1, panel B). Participants responded using their index finger of each hand. In the proximal stimulus-hand condition, each index finger was located on either the left or right loudspeaker. The distal stimulus-hand condition remained as in Experiment 1. In both conditions, participants placed their head on a chinrest attached to the desk, which was positioned approximately 50 cm in front of the monitor.

#### Procedure & data analysis

Except for the fact that the participants placed their hands on the loudspeakers in the proximal stimulus-hand condition, and placed their head onto a chinrest, the procedure and data analysis were kept identical to Experiment 1.

### Results

Results are presented in Fig. 2.

#### RT and ER analyses

For the analyses of RT, all erroneous trials (3.8%) and trials with RT < 200 ms (0.0%) or > 3 SDs (1.3%) of the individual condition mean were removed from further analysis.

The rmANOVA with the within-subjects factors Congruency and Stimulus-hand proximity on RTs revealed no overall Stroop Effect (9 ms ± 5 ms), as the factor Congruency was not significant, *F*(1, 38) = 1.08, *p* = .306, η^2^_p_ = 0.03. Thus, RTs did not differ between incongruent trials (676 ms ± 19 ms) and congruent trials (668 ms ± 17 ms). Further, the factor Stimulus-hand proximity did not affect the overall RTs, *F*(1, 38) = 2.31, *p* = .138, η^2^_p_ = 0.06 (proximal: 682 ms ± 18 ms; distal: 662 ms ± 18 ms). Finally, there was no interaction between the factors Stimulus-hand proximity and Congruency *F*(1, 38) = 0.002, *p* = .966, η^2^_p_ < 0.001, indicating that the Stroop effect was absent for both hand positions (proximal: 8 ms ± 7 ms, distal: 9 ms ± 5 ms) (see Fig. 2, Experiment 2).

ER analysis revealed an overall significant Stroop effect, *F*(1, 38) = 37.740, *p* < .001, η^2^_p_ = 0.50 (3.1% ± 0.3%). Thus, ERs were larger in incongruent trials (5.2% ± 0.4%) compared to congruent trials (2.3 ± 0.3%). However, neither the ER differed according to hand position, *F*(1, 38) = 3.37, *p* = .074, η^2^_p_ = 0.08 (proximal: 3.4% ± 0.4%; distal: 4.1% ± 0.4%), nor was the Stroop effect modulated by the hand position, indicated by a non-significant two-way interaction between Stimulus-hand proximity and Congruency *F*(1, 38) = 1.09, *p* = .303, η^2^_p_ = 0.03 (Stroop effect = proximal: 2.6% ± 0.4%; distal: 3.3% ± 0.4%).

#### Exploratory pooled analysis of selected voice files of experiment 1 & 2

Descriptively, the Stroop effect in RTs observed in Experiment 1 (11 ms, *p* = .048) and in Experiment 2 (9 ms, *p* = .306) was overall relatively small in comparison to previous studies, in which larger effects in similar auditory gender identification Stroop tasks were reported (Stroop effects: 59 ms to 79 ms; see Green & Barber, 1981, 1983)^2^. Irrespective of the reason for the small Stroop effect in Experiment 1 and 2, the missing modulation of the auditory Stroop effect by the stimulus-hand proximity might be due to the small effect size. In other words, the predicted reduction in the Stroop effect may be hard to observe if no reliable Stroop effect is present initially. We therefore addressed this concern in an exploratory pooled data analysis of Experiment 1 and 2 including only voice files that elicited sufficient Stroop interference.

For this, the Stroop effect in Experiment 1 and 2 was inspected for each individual speaker voice. To identify if the Stroop effect differed for each individual voice file, the data were plotted according to congruency for each Experiment (see Appendix A Figure 4). In the RT data, three voice files were identified that showed a reversed congruency effect, i.e. smaller RTs in incongruent trials in comparison to congruent trials. We repeated the rmANOVA on the pooled data of Experiment 1 and 2 including only the 3 voice files showing a positive Stroop effect and tested if this existing Stroop effect was modulated by the Stimulus-hand proximity.

As expected, the pooled filtered data analysis of RTs revealed a large overall Stroop effect (48 ms ± 4 ms), indicated by a significant effect of the factor Congruency *F*(1, 70) = 58.97, *p* < .001, η^2^_p_ = 0.46 (see Fig. 2, Pooled Data). Thus, RTs were larger in incongruent trials (676 ms ± 13 ms) compared to congruent trials (628 ms ± 11 ms). However, neither did the hand position affect the overall RTs, *F*(1,70) = 1.47, *p* = .230, η^2^_p_ = 0.02 (proximal: 658 ms ± 12 ms; distal: 646 ms ± 13 ms), nor did we observe a significant interaction between the factors Stimulus-hand proximity and Congruency *F*(1, 70) = 0.27, *p* = .606, η^2^_p_ < 0.01, indicating that the Stroop effect did not differ according to the hand positions (proximal: 46 ms ± 5 ms, distal: 50 ms ± 5 ms).

The pooled filtered data analysis of the ER revealed an overall significant Stroop effect, *F*(1, 70) = 50.60, *p* < .001, η^2^_p_ = 0.42 (3.9% ± 0.3%). Thus, ERs were larger in incongruent trials (6.0% ± 0.5%) compared to congruent trials (1.7 ± 0.2%). Further, the ER differed according to hand position, *F*(1, 70) = 4.10, *p* = .046, η^2^_p_ = 0.06. Participants committed slightly less errors in the proximal stimulus-hand condition (3.3% ± 0.4%) in comparison to the distal stimulus-hand condition (4.1% ± 0.4%). However, most importantly for this study, the Stroop effect was not modulated by the hand position, indicated by the absence of an interaction between the factors Stimulus-hand proximity and Congruency, *F*(1, 70) = 1.208, *p* = .275, η^2^_p_ = 0.02 (Stroop effect = proximal: 3.5% ± 0.5%; distal: 4.3% ± 0.5%).

### Discussion

Experiment 2 was essentially a replication of Experiment 1, except that in the proximal stimulus-hand condition, the participants’ hands were clearly visible (Davoli et al., 2010; Ellinghaus et al., 2023; Fischer & Liepelt, 2020; Liepelt & Fischer, 2016; Wang et al., 2014, 2021; Yan et al., 2024). The absence of a modulation of the Stroop effect by stimulus-hand proximity in Experiment 2 replicated the findings of Experiment 1. This finding indicates that the non-visibility of the response hands in the proximal stimulus-hand condition was not responsible for the missing near-hand modulation in Experiment 1. An exploratory inspection of the data revealed that three voice files produced an inverted Stroop effect. Even after pooling the data from Experiments 1 and 2 and restricting the analysis to voice files displaying a large overall positive Stroop effect, no near-hand modulation of the Stroop effect was observed. The results of the analyses of the pooled filtered data did not support the assumption that the absence of the near-hand effect in Experiments 1 and 2 was due to low statistical power or inadequate stimulus material. However, the observed interference in the auditory Stroop task suggests that semantic processing is generally intact, and that we did not find evidence of changes in cognitive control or impaired semantic processing between the proximal and distal stimulus-hand conditions in the present auditory Stroop task.

## Experiment 3

The null findings of Experiments 1 and 2 may indicate that previously reported near-hand effects in semantic processing are limited to the visual modality. It has been suggested that the stimulus-hand proximity results in poorer semantic processing of visual stimuli due to increased processing of spatial attributes, i.e., the spatial relationship between the stimulus and the nearby response hand (Davoli et al., 2010). However, this possibility was precluded by the auditory stimulus presentation in Experiments 1 and 2. Experiment 3 therefore tested whether near-hand effects are bound to the processing of spatial stimulus features in particular. To emphasize the importance of stimulus location in relation to the corresponding hand, we reintroduced a spatial task feature into the current design – this time as an irrelevant spatial characteristic of the auditory stimuli. In other words, the task-irrelevant semantic feature (i.e., the spoken word) of our paradigm was replaced with a task-irrelevant spatial feature (i.e., the location of the voice). This essentially converted the auditory Stroop task into an auditory Simon task, using the same stimulus material and response setup as in Experiment 2. This transformation enabled us to draw conclusions about the role of spatial stimulus characteristics in the auditory near-hand effect, and also provided a conceptual replication of Wang et al.’s (2021) study.

In Experiment 3, one of the Stroop-congruent voice files from Experiments 1 and 2 was used for each gender, for example, one male voice said ‘*man’* and one female voice said ‘*woman’*. These voices were presented through either the left or the right loudspeaker. Participants were instructed to respond with left and right key presses to the gender of the voice they heard (i.e., male or female) and to ignore the spatial location of the stimulus presentation^3^. This transformed the auditory Stroop task of Experiments 1 and 2 into an auditory Simon task, in which the spatial presentation of the stimulus was irrelevant. The re-emergence of a near-hand effect in this design would suggest stronger spatial coupling between stimuli and responses in near-hand space. In line with recent research (Wang et al., 2021), we predicted increased interference in the proximal than the distal stimulus-hand condition.

### Method

The design, hypotheses, and analysis plans of Experiment 3 were preregistered (AsPredicted #140646, https://aspredicted.org/75bg-78gr.pdf).

#### Participants

A new sample of 40 participants was recruited. One participant committed an ER of more than 3 SDs over the sample mean. This participant was identified as an outlier and removed from further analyses. Thus, the final sample consisted of 39 participants (female: 27, gender-divers: 1, right-handed: 38, ambidextrous: 1; age: 24 years ± 2 years, min: 19 years, max: 33 years). All participants reported sufficient knowledge of the German language and normal hearing. The study was conducted in accordance with the guidelines of the 1964 Declaration of Helsinki and those of the German Psychological Society. Participants provided informed consent prior to the experiment. Before the experimental session began, each participant was informed about the study’s purpose and procedure. Participants were compensated with either course credits or a payment of €8.

#### Stimuli & apparatus

The stimuli and apparatus were the same as in Experiment 2, except that only the Stroop-congruent version of one female (saying *woman*) and one male voice file (saying *man*) from the previous experiments were used as stimuli.

#### Procedure

Male and female voices were presented equally often through the left and the right loudspeakers. Participants were instructed to identify the gender of the speaker as quickly and accurately as possible, while ignoring the spatial location of the voice. A stimulus was considered congruent if the voice location matched the response side (e.g., a female voice presented on the left side with a response for a female voice on the left side), and incongruent if the voice location mismatched the assigned response side (e.g., a female voice presented on the right side with a response for a female voice on the left side). The Simon effect was defined as the difference between incongruent and congruent trials. The experimental structure (i.e., the number of trials in the practice and test blocks, the proportion of congruent trials, and the use of feedback) was kept the same as in Experiments 1 and 2. The condition order and S-R mapping were counterbalanced.

#### Data analysis

A 2 (Stimulus-hand proximity: proximal, distal) × 2 (Congruency: congruent, incongruent) within-subjects repeated measures analysis of variance (rmANOVA) was applied on RTs and ERs. The analyses were conducted with the software RStudio (Version 2023.09.1, Posit Software, PBC). The rmANOVA was conducted by using the rstatix package (Version 0.7.2) (Kassambara, 2023). The results are presented in mean ± standard error of the mean. For the analyses of RT all erroneous trials (2.0%) and trials with RTs < 200 ms (0.0%) or > 3 SDs (0.0%) of the individual condition mean were removed from further analysis.

### Results

**Fig. 3:**
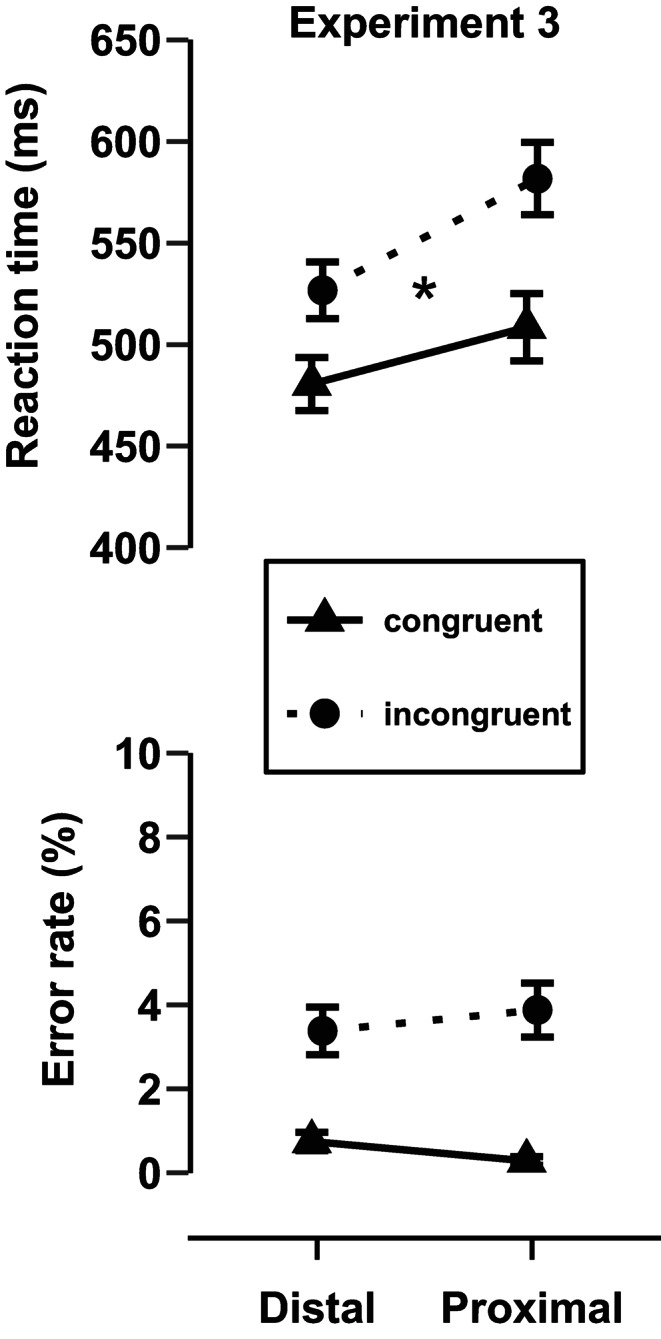
Mean reaction times and error rates as a function of hand position and congruency in Experiment 3. Error bars represent standard errors of the mean.

**Fig. 4:**
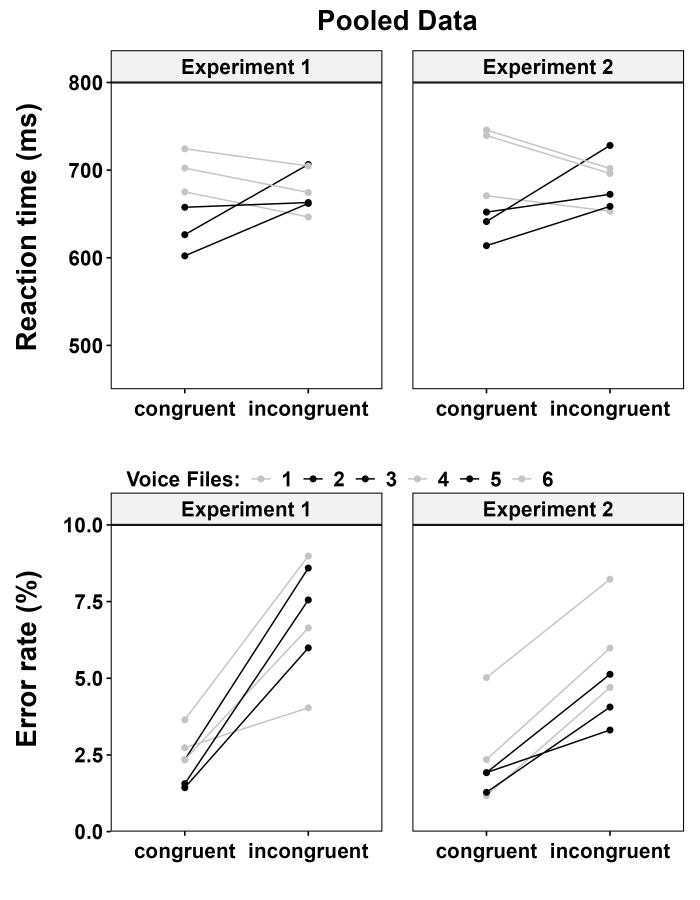
Mean reaction times and error rates as a function of voice file and congruency in Experiments 1 and 2. Voice files 1, 4, and 6 (gray lines) showed a reversed Stroop effect in mean reaction times

Results are presented in Fig. 3.

#### RT and ER analyses

The rmANOVA including the within-subjects factors Congruency and Stimulus-hand proximity on RTs revealed an overall Simon effect (60 ms ± 3 ms), as the factor Congruency was significant, *F*(1, 38) = 161.43, *p* < .001, η^2^_p_ = 0.81. Thus, RTs were larger in incongruent trials (554 ms ± 12 ms) compared to congruent trials (495 ms ± 11 ms). Further, the hand position affected the overall RTs, *F*(1,38) = 20.91, *p* < .001, η^2^_p_ = 0.36 (proximal: 545 ± 13 ms; distal: 504 ± 10 ms), i.e., participants responded generally slower in the proximal compared to the distal stimulus-hand condition. Most importantly, the Simon effect was modulated by the hand position, indicated by a significant interaction between Stimulus-hand proximity and Congruency *F*(1, 38) = 29.55, *p* < .001, η^2^_p_ = 0.44. The Simon effect was larger in the proximal than in the distal stimulus-hand condition (Simon effect = proximal: 73 ms ± 4 ms, distal: 46 ms ± 4 ms).

Analysis of ERs revealed an overall significant Simon effect, *F*(1, 38) = 37.78, *p* < .001, η^2^_p_ = 0.50 (3.1% ± 0.3%). ERs were larger in incongruent trials (3.6% ± 0.4%) compared to congruent trials (0.5 ± 0.1%). However, neither did the ER differ according to hand position, *F*(1, 38) = 0.01, *p* = .947, η^2^_p_ < 0.001 (proximal: 2.1% ± 0.4%; distal: 2.1% ± 0.3%), nor was the Simon effect modulated by the hand position for the ERs, as Stimulus-hand proximity and Congruency did not interact, *F*(1, 38) = 2.50, *p* = .122, η^2^_p_ = 0.06 (Simon effect = proximal: 3.6% ± 0.4%; distal: 2.6% ± 0.4%).

### Discussion

In Experiment 3, we replaced the task-irrelevant semantic feature of the stimulus with a task-irrelevant spatial feature. Using the same stimulus and response setup as in Experiment 2, we transformed the auditory Stroop task into an auditory Simon task. Consistent with the assumption that close spatial proximity between the stimulus and the response in the proximal stimulus-hand condition might lead to stronger activation of the corresponding effector, the results revealed a significant near-hand modulation of the spatial S-R interference effect. More precisely, a larger auditory Simon effect was observed in the proximal stimulus-hand condition (73 ms) than in the distal stimulus-hand condition (46 ms). These results confirm that spatial proximity between the stimulus and the response in near-hand space leads to greater interference in the auditory modality, similar to what has been observed in the visual modality (Liepelt & Fischer, 2016; Wang et al., 2014, 2018; Yan et al., 2024).

## General discussion

The present study aimed to determine whether the reduced Stroop effect observed when visual stimuli are presented in near-hand space (Davoli et al., 2010) also occurs with auditory Stroop stimuli presented close to the hands. For S-R conflict tasks such as the Simon task, it has been shown that stimulus-hand proximity modulates the interference effect for both visual and auditory stimulus presentations. It has been suggested that this modulation is based on the spatial proximity between the stimulus and the response in the proximal stimulus-hand condition. This proximity may increase the coupling between the spatial stimulus and response features (Wang et al., 2014, 2021), facilitating a response activation that is specific to the near hand. In S-S conflict tasks such as the Stroop task, Stroop interference is reduced in near-hand space when a visual stimulus is presented (Davoli et al., 2010). However, it remained unclear whether this finding is due to impaired semantic processing, facilitated processing of spatial task attributes, or increased involvement of cognitive control.

Three experiments were conducted in which participants responded to auditory stimuli. Stimulus-hand proximity was manipulated by positioning the hands either near (proximal stimulus-hand condition) or far from the stimuli (distal stimulus-hand condition). In Experiment 1, the auditory stimuli were presented via headphones. In the proximal stimulus-hand condition, the participants’ hands were positioned outside their visual field, at the headphones. In Experiments 2 and 3, the stimuli were presented through loudspeakers positioned in front of the participants. The participants’ hands were placed on the loudspeakers, positioning both the stimuli and the hands within their visual field, as in previous studies (Davoli et al., 2010; Ellinghaus et al., 2023; Fischer & Liepelt, 2020; Liepelt & Fischer, 2016; Wang et al., 2014, 2021; Yan et al., 2024). In Experiments 1 and 2, stimulus-hand proximity did not affect the auditory Stroop effect. Although the auditory Stroop effect was evident in ER in both experiments, its size in RTs was relatively small (failing to reach statistical significance in Experiment 2), which may have limited the potential for modulation by stimulus-hand proximity. An exploratory pooled-data analysis of both experiments, restricted to voice files with large overall Stroop effects for RTs, also did not reveal an influence of stimulus-hand proximity on the auditory Stroop effect.

The finding of comparable auditory Stroop effects in distal and proximal stimulus-hand conditions is inconsistent with differential involvement of cognitive control, which would predict differences in auditory Stroop effects between stimulus-hand proximity conditions. Furthermore, the findings of Experiments 1 and 2 do not suggest that presenting stimuli near the hands generally impairs semantic processing or heightens attention in the near-hand space for auditory stimuli. This is in line with a recent study, that found no modulation of the near-hand effect on syntactic analysis in sentences (Grossi et al., 2021; but see Experiment 1, Davoli et al., 2010). Experiment 3 provided evidence that near-hand effects are not limited to the visual modality and can also emerge when task-irrelevant spatial stimulus attributes are introduced in an auditory task. That is, in Experiment 3, the task-irrelevant semantic feature of the auditory stimulus (i.e., gender in Experiments 1 and 2) was converted into a task-irrelevant spatial stimulus feature (i.e., location). As a result, the stimulus-hand proximity significantly increased the auditory interference effect.

The increased relevance of spatial stimulus attributes in near-hand space is consistent with existing evidence of reduced interference effects in visual S-S conflict tasks (Davoli et al., 2010; Davoli & Brockmole, 2012; Englert & Wentura, 2016) and increased interference effects in visual and auditory S-R spatial conflict tasks (Liepelt & Fischer, 2016; Wang et al., 2014, 2018; Yan et al., 2024). A bias towards processing of physical spatial attributes in near-hand space as suggested by Davoli et al. (2010) aligns with existing evidence of slower attentional reallocation in the near-hand space (Abrams et al., 2008), and the role of spatial attention in reading (Gabay et al., 2020; Vidyasagar & Pammer, 2010). Since objects near the hands are potential interaction canditates, it is therefore plausible that stimulus processing near the hands would be biased towards an objects’ low-level physical properties, such as location. This is particularly evident in S-R conflicts of the Simon task. In these conflicts, the location of the stimulus is a salient yet task-irrelevant spatial stimulus attribute that is spatially referenced to a corresponding response (Dolk et al., 2013; Hommel, 1993). It has been argued that the close distance between stimulus and response in the proximal stimulus-hand condition facilitates the coupling between a stimulus and its associated motor response (Wang et al., 2014). Consequently, the increased relevance of spatial attributes in near-hand space may lead to stronger response activation of the spatially compatible response, resulting in larger interference effects, as observed in Experiment 3 of the present study. Since near-hand effects have been observed with both visual and auditory stimuli in the Simon task, the modality of the stimuli may be less important for near-hand effects in spatial S-R conflicts.

Recent discussions have centred on the robustness versus fragility of near-hand effects in general (Dosso & Kingstone, 2018). While some studies have reported failed replications (Andringa et al., 2018; Dosso & Kingstone, 2018), others have replicated earlier findings on near-hand effects, showing faster detection rates for simple targets (McManus & Thomas, 2018; Reed et al., 2006, 2010; Sun & Thomas, 2013) and for changes in stimulus color and orientation (Bridgeman & Tseng, 2011; Kelly & Brockmole, 2014) in near-hand space than in far-hand space (Agauas et al., 2020). In light of the ongoing debate, our study provides valuable insights by conceptually replicating and extending previous findings of enhanced spatial interference in the near-hand space for auditory stimuli. Whereas Wang et al. (2021) demonstrated an increase in spatial interference related to the near hand using a tone frequency categorization Simon task, our results show that this effect also occurs in a voice gender categorization Simon task. This supports earlier evidence of a larger Simon effect in the near-hand space within the visual modality (Liepelt & Fischer, 2016; Wang et al., 2014, 2018; Yan et al., 2024). However, we were unable to extend the near-hand modulation of the visual Stroop effect (Davoli et al., 2010) to the auditory domain. This emphasizes the importance of replicating and extending near-hand effects in different sensory modalities, in order to clarify the conditions under which these effects reliably occur. Future studies may investigate the extent to which the effects of stimulus-hand proximity depend on the characteristics of cognitive processing. In particular, the nature of the interactions between stimulus-hand proximity and cognitive control in more complex situations remains to be determined (Liepelt & Fischer, 2016; Weidler & Abrams, 2014).

### Conclusion

This study is the first to investigate the impact of stimulus-hand proximity on semantic processing in auditory stimuli using the Stroop task. Unlike as in visual modalities, we found no evidence that the proximity between the stimulus and the hand modulates semantic processing for auditory stimuli. However, as in visual modalities, stimulus-hand proximity enhanced interference in auditory spatial S-R conflict tasks. These results suggest that the near-hand effect appears to be robust across sensory stimulus modalities when processing physical spatial attributes in S-R conflict tasks.

## Data Availability

The raw data are publicly available at PsychArchives, DOI: 10.23668/psycharchives.21158.

## Appendix A

See Fig. 4

## Footnotes

1 We thank the authors for providing the stimulus material.

2 Larger Stroop effects in these studies may be due to differences in the applied S-R setting. For example, labelling the response keys with the semantic meaning of the applied stimuli (Green & Barber, 1981) or vocally responding with words that matched or mismatched the semantic meaning of the applied stimuli (Green & Barber, 1983) may have boosted the Stroop effect in their studies.

3 Because of using Stroop congruent voice files only, we had no control whether participants truly responded to the speakers’ gender or to the spoken word. Both features, however, map redundantly onto the required response, which, in the worst case, might reduce the size of the auditory Simon effect.
